# Use of Tumguide® in the Insertion of a Nasogastric Tube Into the Stomach of a Patient With Creutzfeldt-Jakob Disease in a Nursing Home: A Case Report

**DOI:** 10.7759/cureus.52082

**Published:** 2024-01-11

**Authors:** Tomoya Iida, Yumi Kodama, Kazue Kogina

**Affiliations:** 1 General Practice, Sapporo Home Care Clinic Soyokaze, Heiikukai Medical Corporation, Sapporo, JPN; 2 Nursing, Faculty of Health Sciences, Hokkaido University of Science, Sapporo, JPN

**Keywords:** tumguide®, biologically transparent illumination, home care, creutzfeldt-jakob disease, nasogastric tube

## Abstract

A 77-year-old woman exhibited a rapid progression of dementia and declining physical function and, over a period of about four months, reached a state of akinetic mutism. A final diagnosis of Creutzfeldt-Jakob disease (CJD) was made. A nasogastric tube was inserted into the stomach, and then it was confirmed on X-ray that the end of the tube was in the correct position. She was discharged to a nursing home, where she received home medical care after discharge. One month after the nasogastric tube insertion, Tumguide® was used to assist in replacing the tube at this home. In home care settings where an X-ray machine may not be available, Tumguide® may assist with nasogastric tube insertion.

## Introduction

When artificial nutrition is being considered for patients with sequelae of cerebral infarction or advanced dementia, who have difficulty eating by mouth, enteral feeding is common [1]. In most cases, the administration route for tube feeding is via a nasogastric tube or gastrostomy, but the indications depend on a range of factors, including the patient’s overall condition and disease, where they are being cared for, and their prognosis.

When a nasogastric tube is inserted into the stomach of a patient in a hospital, this is usually done under plain X-ray observation to ensure that the end of the tube is not inserted anywhere other than into the stomach [2]. However, in home care settings, an X-ray machine may not always be available.

Tumguide® (Neuroceuticals Inc., Tokyo, Japan) is a handy system for checking nasogastric tube tip placement. It consists of a Tumguide® light source and Tumguide® fiber. The light source utilizes proprietary light-condensing technology to emit a red light-emitting diode (LED) that is highly biologically permeable for use as biologically transparent (BT) illumination [3]. To use this system, the light source is connected to a fiber inserted into the nasogastric tube, and the lit-up tip is passed through the esophagus and into the stomach, where its location is visible from outside the body. This has the advantage that a nasogastric tube can be placed without using X-rays [4].

We present a patient, treated for Creutzfeldt-Jakob disease (CJD), who underwent a nasogastric tube insertion with Tumguide® while in a nursing home. It is the first report of the use of Tumguide® for nasogastric tube insertion in a home care setting.

## Case presentation

The patient was a 77-year-old woman, with a height of 154 cm and a weight of 54 kg. Her medical and family history were unremarkable, and she had never lived outside Japan. She lived alone in a detached house, and the persons closest to her were her older son and her younger son with his wife.

In March 2023, she noticed that her sight was becoming poor and visited an ophthalmologist. There were signs of cataracts in both eyes, and she underwent surgery in June 2023, but there was little improvement. The next month, she developed obvious visual and auditory hallucinations, became violent, and attempted self-harm, so she was sent to a psychiatric hospital for her own protection. As the amount she was eating decreased while she was hospitalized, a nasogastric tube was inserted into her stomach, and enteral feeding was started after chest and abdominal X-rays showed no abnormalities. Lewy body dementia was initially suspected. A dose of 4.5 mg rivastigmine patch treatment was initiated and increased to 9 mg after four weeks. However, her symptoms continued to worsen, and she was transferred to a general hospital under the care of an attending physician in the Department of Neurology.

At the time of the hospital transfer, she was akinetic and mute, exhibiting signs of generalized myoclonus. Laboratory tests revealed hypoproteinemia (6.0g/dL (6.7-8.3 g/dL)) and hypoalbuminemia (3.2g/dL (3.8-5.2 g/dL)) without other abnormalities (Table 1).

**Table 1:** Laboratory test findings.

Items	Values	Unit	Reference ranges		Items	Values	Unit	Reference ranges	
WBC	6,300	/μL	4,000-8,000		Na	137	mEq/L	137-147	
RBC	418	×10^6^/μL	380-500		K	3.8	mEq/L	3.5-5.0	
Hb	12.5	g/dL	12.0-16.0		Cl	100	mEq/L	98-108	
Plt	26.7	×10^4^/μL	12.0-40.0		Ca	8.5	mg/dL	8.4-10.2	
TP	6.0	g/dL	6.7-8.3		T-chol	173	mg/dL	130-220	
Alb	3.2	g/dL	3.8-5.2		TG	76	mg/dL	30-149	
T-Bil	0.6	mg/dL	0.2-1.2		fT3	2.5	pg/mL	1.7-3.7	
AST	29	U/L	8-38		fT4	1.6	ng/dL	0.7-1.5	
ALT	19	U/L	4-44		TSH	0.9	μIU/mL	0.4-4.9	
LDH	204	U/L	120-245		CRP	0.1	mg/dL	≦0.3	
ALP	79	U/L	38-113		HBs-Ag	(-)		(-)	
AMY	60	U/L	37-125		HCV-Ab	(-)		(-)	
BUN	9.5	mg/dL	8.0-20.0		HIV-Ab	(-)		(-)	
Cre	0.5	mg/dL	0.5-0.9		FBS	90	mg/dL	70-110	

**Figure 1:**
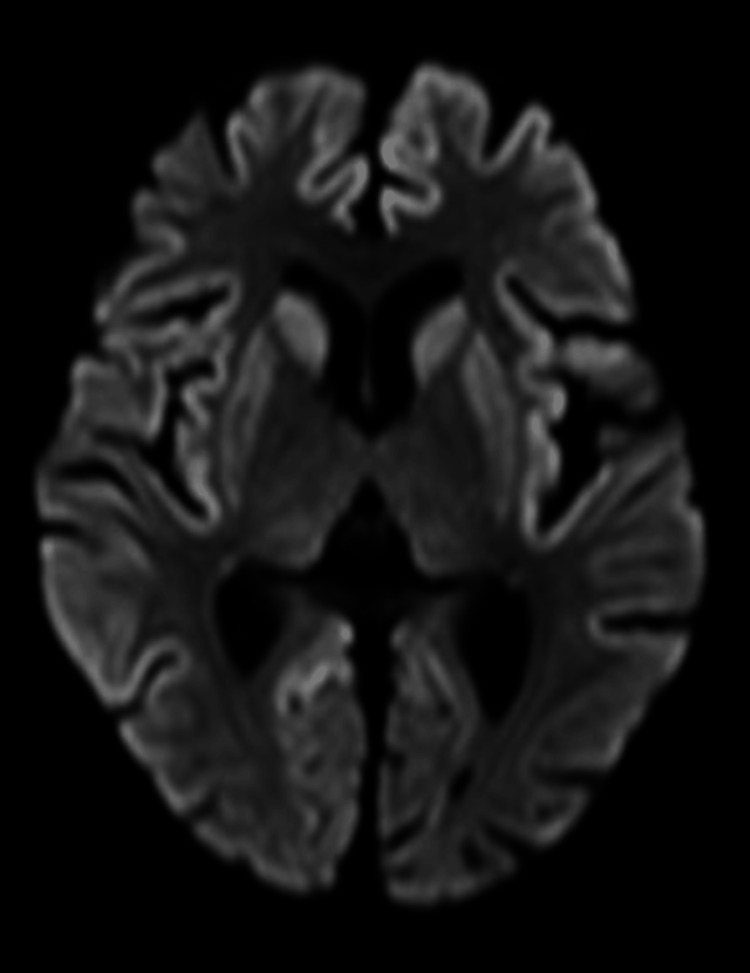
Brain MRI findings. Brain MRI revealed mild atrophy of the cerebrum and high signal levels in the cerebral cortex and basal ganglia on DWI.

Chest and abdominal X-rays showed no abnormalities, except for the presence of a nasogastric tube. Brain MRI revealed mild atrophy of the cerebrum and high signal levels in the cerebral cortex and basal ganglia on DWI (Figure 1).

A periodic synchronous discharge (PSD) was evident on electroencephalography, and cerebrospinal fluid tests revealed positive for 14-3-3 protein and negative for a real-time quaking-induced conversion (RT-QuIC) assay. In September 2023, she was finally diagnosed with CJD with a life expectancy of around six months.

Although she was diagnosed with CJD, the possibility of a variant of this disorder was not completely excluded, and there was some hesitancy in performing a gastrointestinal endoscopy to create a gastrostomy. It was, accordingly, decided that enteral feeding would be continued via a nasogastric tube.

While the patient was hospitalized, visiting was strictly limited to prevent the spread of COVID-19. In the process of deciding where she should be subsequently cared for, her family requested that she would be discharged to a facility where visits would be possible. It was, therefore, decided to discharge her to a nursing home and to initiate home medical care after she was discharged.

After her discharge to an environment in which her family was able to visit, her general condition remained stable. One month after nasogastric tube insertion at the previous hospital, tube replacement was considered. As the facility did not possess a portable X-ray scanner, the staff consulted the family on whether they would prefer the tube to be changed in a visit to the hospital or by using Tumguide® in the facility, and they chose the latter.

**Figure 2:**
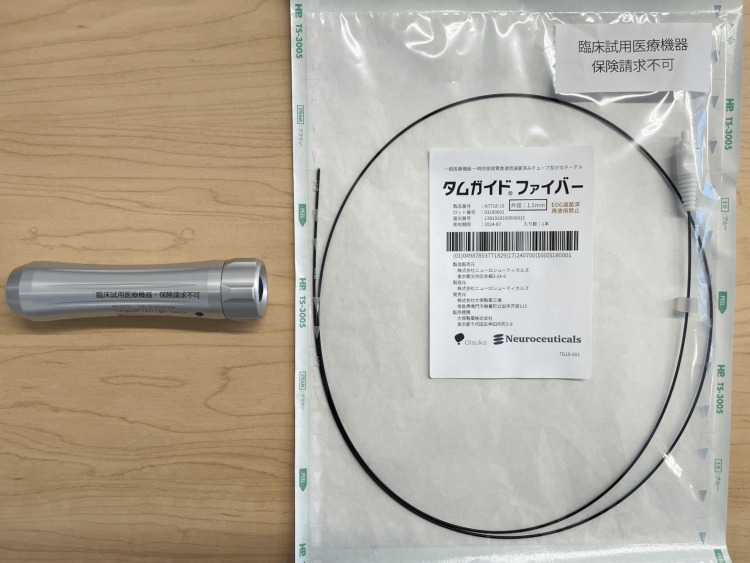
Main components of Tumguide®.

Tumguide® consists of a Tumguide® light source (Figure 2, left) and a disposable Tumguide® fiber (Figure 2, right).

**Figure 3:**
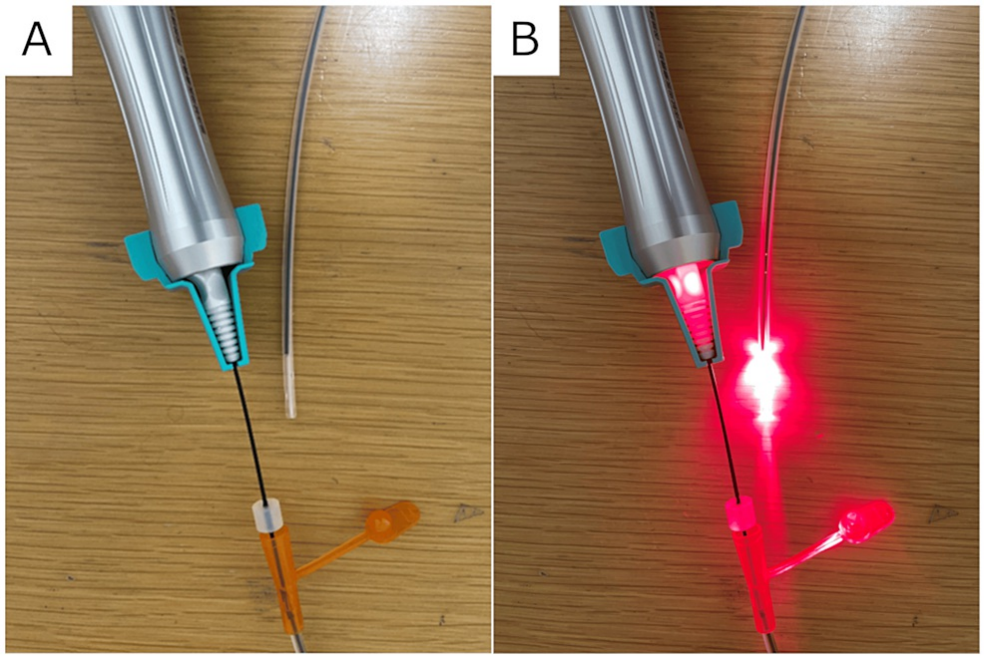
Tumguide® system.

The nasogastric tube that had been inserted in the previous hospital was removed. The Tumguide® fiber (external diameter of 1.5 mm) was coated with medical-grade lubricating jelly and inserted into a new nasogastric tube (12Fr x 120 cm; TOP Co., Tokyo, Japan) and anchored with a stopper at a position sufficiently far from the end of the tube to ensure that the tip would not poke out beyond it. The Tumguide® fiber was connected to a Tumguide® light source, and a stopper was fitted to prevent the two from coming apart (Figure 3A). The power was turned on, and it was confirmed that the BT light was blinking at the tip (Figure 3B).

**Figure 4:**
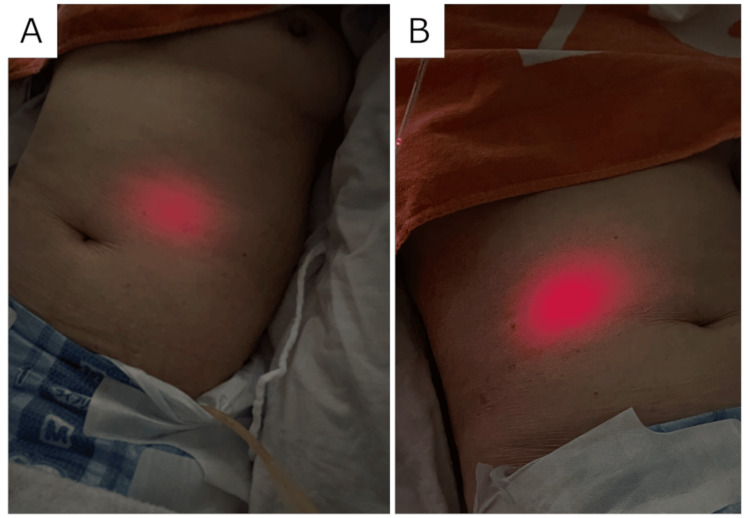
Findings that the tip of the Tumguide® fiber exists in the stomach.

The room lights were turned off, the curtains were drawn, and the nasogastric tube was inserted gently and smoothly in a supine position. The BT light was observed above and to the left of the umbilicus, indicating that the tip had reached the inside of the stomach (Figure 4A). At this point, the insertion length was 50 cm, and when the tube was pushed further in, at an insertion length of 55 cm, the BT light was observed above and to the right of the umbilicus, indicating that the tip had moved to the pyloric side of the stomach and suggesting that the end of the nasogastric tube was within the stomach (Figure 4B).

The nasogastric tube was fixed at the 50 cm position, after which the Tumguide® fiber was withdrawn. When air was injected, a bubbling sound was heard in the stomach, and around 20 mL of clear yellow fluid with the appearance of gastric fluid was aspirated from the nasogastric tube.

After this exchange, only water was initially administered, and as this was found to be unproblematic, enteral nutrition agents were then administered. Three weeks after the tube was exchanged, the patient’s course was uneventful, and we intend to use Tumguide® again for nasogastric tube exchanges at the facility in the future.

## Discussion

We treated a patient with CJD who underwent nasogastric tube insertion with Tumguide® in a nursing home. This is the first case report of the use of Tumguide® for nasogastric tube insertion in a home care setting.

Nasogastric tubes are useful for short-term enteral nutrition, but when long-term enteral nutrition is required, percutaneous endoscopic gastroscopy (PEG) nutrition is the usual choice. However, CJD is a prion disease, and although sporadic, the infectious prions are almost completely localized to the eyes and central nervous system, and the risk of infection due to gastrointestinal endoscopy is regarded as negligible [5]. In variant CJD, the prions are present, at a comparatively high level, in the lymphatic tissue, including the tonsils and Peyer’s patches in the intestines. Even after high-level disinfection, the possibility of residual prions cannot be excluded [6,7]. Although the probability of encountering variant CJD in Japan is now extremely low, the possibility could not be entirely excluded in this case. Given the resultant hesitancy about performing a gastrointestinal endoscopy for a gastrostomy, it was decided to continue enteral feeding via a nasogastric tube.

Nasogastric tubes are comparatively easy to use for delivering enteral nutrition. However, if they are mistakenly inserted into the trachea and bronchi, subsequent nutrient administration may cause extremely serious complications. In a previous study of 740 nasogastric tube insertions, the tube was mistakenly inserted into the trachea and bronchi in 14 cases (1.9%), two (0.3%) of which resulted in the development of fatal complications [8].

The use of a laryngoscope has been reported to be useful as per the Japan Medical Safety Agency's guidelines for nasogastric tube insertion [9]. Although it can prevent insertion into the trachea, it cannot determine the position of the tip of the gastric tube. It has been customary to use simple methods of confirming the position of the tip of the tube after nasogastric tube insertion, including listening for gastric bubbling sounds and aspirating gastric contents, but these confirmation methods are far from safe and do not match the efficacy of X-ray guidance [2]. The sensitivity of listening for gastric bubbling sounds was 79%, while the specificity was 61%. Moreover, if an aspirate could be obtained, the results of pH measurements showed a sensitivity of 78.4% and a specificity of 85.7% [10].

In home medical care settings, however, the number of facilities that have a portable X-ray scanner is limited. Under these circumstances, Tumguide® is a useful alternative. When Tumguide® was in development, a study was carried out to compare the visible position of the BT light from Tumguide® with the position of the end of the tube on X-rays in 102 hospitalized adult patients requiring general anesthesia. When the BT light was visible in the upper abdominal region, the end of the nasogastric tube was always inside the stomach (87/87: positive predictive value 100%), and when the end of the tube was confirmed not to be inside the stomach on X-ray, the BT light was never visible (4/4: specificity 100%). However, the BT light was not always visible when the end of the tube was confirmed to be inside the stomach on X-ray (87/98: sensitivity 89%) [11]. These results suggest that, when the BT light is visible in the upper abdominal region, it is extremely likely that the end of the nasogastric tube is inside the stomach, and it can be used with appropriate precaution. There are various reasons why the BT light may not be visible, even when the nasogastric tube is inside the stomach. These include situations where its end is positioned tangentially to the gastric wall because of the presence of multiple organs, including the left lobe of the liver and the transverse colon on the abdominal side of the stomach, and because of food residue within the stomach [11]. When the BT light is not visible, the end of the nasogastric tube may be outside the stomach, such as inside a lung, and the tube should not be used.

Tumguide® has the advantage over X-ray scanning in that it does not expose either the patient or medical staff to radiation. Another method of checking the position of the nasogastric tube that also does not involve X-ray exposure is ultrasound scanning. A recent systematic review of 14 studies, involving a total of 1,812 adult subjects, reported that this method has a sensitivity of 96% and specificity of 91% [12]. Although this is a useful method, it is unable to detect misplacement in a certain number of cases and requires a high level of proficiency. A further confirmation method is the use of a capnometer to measure the partial pressure of carbon dioxide in exhaled air. A systematic review that analyzed 13 studies and involved a total of 1,541 adult subjects reported that this technique has a sensitivity of 96% and a specificity of 99% [13]. Thus, Tumguide® like a capnometer is considered to be a useful option for inserting a nasogastric tube safely.

One of the problems with the Tumguide® system is the cost. Even though the Tumguide® light source device costs 400,000 yen and the disposable Tumguider® fiber costs 2,300 yen, the procedure fee can only be calculated at 1,800 yen. For Tumguide®, in order to spread the technology further, an improvement in this regard is urgently desired.

This is the first case report of the use of Tumguide® for nasogastric tube insertion in a home medical care setting. There has been a report of its use for hospitalized patients in Japan [14]. Tumguide® was used for two hospitalized patients with COVID-19. In one, the nasogastric tube placement was successful, and in the other, the BT light was not observed. Tumguide® may also be useful from an infection control perspective as it enables the nasogastric tube placement to be completed at the bedside. Following COVID-19, the possibility of another novel infectious disease causing a pandemic that requires isolation in the near future cannot be ruled out, and the sphere of use of Tumguide® is expected to grow.

## Conclusions

We treated a patient with CJD who underwent nasogastric tube insertion with Tumguide® in a nursing home. This is the first case report of the use of Tumguide® for nasogastric tube insertion in a home care setting. Tumguide® may be useful for nasogastric tube insertion in home care settings where it may not be possible to confirm the position of the end of the nasogastric tube by X-ray. The limitation of the Tumguide® system is the cost. Since it is a new and useful technique for nasogastric tube insertion, further improvement in the cost is urgently desired.

## References

[1] Sampson EL, Candy B, Jones L (2009). Enteral tube feeding for older people with advanced dementia. Cochrane Database Syst Rev.

[2] Lortie MA, Charbonney E (2016). Confirming placement of nasogastric feeding tubes. CMAJ.

[3] Jagdeo JR, Adams LE, Brody NI, Siegel DM (2012). Transcranial red and near infrared light transmission in a cadaveric model. PLoS One.

[4] Hirano H, Masaki H, Kamada T, Taniguchi Y, Masaki E (2021). Biologically transparent illumination is a safe, fast, and simple technique for detecting the correct position of the nasogastric tube in surgical patients under general anesthesia. PLoS One.

[5] Alvarade CJ, Reichelderfer M (1994). APIC guideline for infection prevention and control in flexible endoscopy. Am J Infect Control.

[6] Will RG, Ironside JW, Zeidler M (1996). A new variant of Creutzfeldt-Jakob disease in the UK. Lancet.

[7] Hill AF, Butterworth RJ, Joiner S (1999). Investigation of variant Creutzfeldt-Jakob disease and other human prion disease with tonsil biopsy samples. Lancet.

[8] Rassias AJ, Ball PA, Corwin HL (1998). A prospective study of tracheopulmonary complications associated with the placement of narrow-bore enteral feeding tubes. Crit Care.

[9] Japan Medical Safety Research Organization (2024). Analysis of death cases related to gastric tube insertion for the purpose of administering nutritional supplements (Content in Japanese) (2018). https://www.medsafe.or.jp/modules/advocacy/index.php?content_id=54, Accessed: January 4, 2024.

[10] Boeykens K, Steeman E, Duysburgh I (2014). Reliability of pH measurement and the auscultatory method to confirm the position of a nasogastric tube. Int J Nurs Stud.

[11] Masaki H, Hirano H, Takahashi J, Kamada T, Masaki E (2023). An improved biologically transparent illumination system that increases the accuracy of detecting the correct position of a nasogastric tube in the stomach. PLoS One.

[12] Peng J, Tang M, Liu LL, Chen WT, Ye QH (2022). Diagnostic accuracy of ultrasonography for detecting gastric tube placement: an updated meta-analysis. Eur Rev Med Pharmacol Sci.

[13] Chau JP, Liu X, Choi KC (2021). Diagnostic accuracy of end-tidal carbon dioxide detection in determining correct placement of nasogastric tube: an updated systematic review with meta-analysis. Int J Nurs Stud.

[14] Okita K (2023). Two cases of nasogastric tube insertion using Tumguide® for isolated hospitalized patients with COVID-19 (content in Japanese). JJSPEN.

